# Predictive simulation of post-stroke gait with functional electrical stimulation

**DOI:** 10.1038/s41598-021-00658-z

**Published:** 2021-11-01

**Authors:** Gilmar F. Santos, Eike Jakubowitz, Nicolas Pronost, Thomas Bonis, Christof Hurschler

**Affiliations:** 1 Laboratory for Biomechanics and Biomaterials, Department of Orthopedics, Hannover Medical School, Hannover, Germany; 2 CNRS LIRIS, Université Claude Bernard Lyon 1, Université de Lyon, Lyon, France

**Keywords:** Biomedical engineering, Computational biophysics, Stroke, Musculoskeletal models, Biological physics

## Abstract

Post-stroke patients present various gait abnormalities such as drop foot, stiff-knee gait (SKG), and knee hyperextension. Functional electrical stimulation (FES) improves drop foot gait although the mechanistic basis for this effect is not well understood. To answer this question, we evaluated the gait of a post-stroke patient walking with and without FES by inverse dynamics analysis and compared the results to an optimal control framework. The effect of FES and cause-effect relationship of changes in knee and ankle muscle strength were investigated; personalized muscle–tendon parameters allowed the prediction of pathologic gait. We also predicted healthy gait patterns at different speeds to simulate the subject walking without impairment. The passive moment of the knee played an important role in the estimation of muscle force with knee hyperextension, which was decreased during FES and knee extensor strengthening. Weakening the knee extensors and strengthening the flexors improved SKG. During FES, weak ankle plantarflexors and strong ankle dorsiflexors resulted in increased ankle dorsiflexion, which reduced drop foot. FES also improved gait speed and reduced circumduction. These findings provide insight into compensatory strategies adopted by post-stroke patients that can guide the design of individualized rehabilitation and treatment programs.

## Introduction

Patients who have suffered a stroke can present with various typical gait abnormalities including drop foot^1^, stiff-knee gait (SKG)^2^, and knee hyperextension^3^, which may lead to an asymmetric gait pattern. Slow gait speed and asymmetry of spatiotemporal, kinematic, and kinetic parameters are also often observed^4,5^. Spatial asymmetry is typically calculated using inter-limb step length asymmetry, but post-stroke patients may walk with longer paretic steps or longer nonparetic steps^6,7^. Impaired paretic propulsion may be related to longer paretic steps^6,8^. Duration of gait phases such as swing time and stance time could be used in the analysis of temporal symmetry, which could be related to vertical ground reaction force (GRF) symmetry and self-selected gait speed^7,9^. The causes of these impairments have been debated, with no real consensus^10,11^. This is likely due to the complexity of different residuals as well as the variable pathologies of stroke patients, which also make it challenging to establish a cause-effect relationship for pathologic changes in the gait of those patients.

The above-mentioned pathologic gait patterns have specific characteristics. Knee hyperextension occurs during the stance phase of gait, when the knee joint extends beyond the neutral anatomic position; a peak knee extension of up to 22° has been reported in stroke patients^3^. Several factors are thought to contribute to knee hyperextension including early calf muscle activity^10^, ankle plantarflexor muscle weakness, flat foot or forefoot landing patterns^3,11,12^, or knee extensor muscle weakness^13^. SKG is characterized by a lack of knee flexion during the swing phase of gait. Possible causes are overactivity of the ankle plantarflexor or rectus femoris muscles in pre-swing, weakened knee flexor muscles^10^, rectus femoris hyperreflexia^14^, low knee flexion velocity^15^, or impaired ankle push-off^2^. In drop foot, patients are unable to achieve dorsiflexion of the foot during the swing phase of gait, which is presumably caused by peroneal nerve paralysis or paresis, ankle dorsiflexor muscle weakness, or ankle plantarflexor muscle overactivity and shortening^1,10,16^.

Rehabilitation of post-stroke patients typically involves muscle strength and robot-assisted gait training^11,17^, the use of orthoses^11^, botulinum toxin injection^18^, and functional electrical stimulation (FES)^19^. An example of FES that has been shown to improve the gait of drop foot patients is the ActiGait device (Ottobock, Duderstadt, Germany), which is an implantable stimulator placed around the peroneal nerve^20,21^. The neuroprosthesis includes a heel switch that differentiates stance and swing phases during the gait cycle. Based on this information, an adjustable pulse is induced within the stimulator, the motor branch of the common peroneal nerve is stimulated, and a timely adequate dorsiflexion is achieved^21,22^. This FES may reduce the risk of falling and increase self-selected gait speed^22,23^.

Inverse dynamics (ID) is a method that is widely used to estimate the joint moments of human gait. As this approach typically depends on known kinematics and GRF, changes in the musculoskeletal system that significantly affect gait patterns cannot be investigated. Although there is a higher degree of complexity in terms of formulating the problem, predictive simulation methods can provide a better understanding of pathologic gait and the effects of treatments as new motion patterns can be predicted. The ability to choose the speed of simulated gait is also a useful feature of predictive simulation, given its influence on kinematic, kinetic, and muscle activity patterns^24,25^. Several predictive simulation approaches have been proposed. One method is based on forward dynamics simulation where the motion is computed via integration. Stable gait patterns have been generated by this method using reflex-based controllers^26^ or neural networks^27^. Gait prediction may also be formulated as an optimal control problem^28,29^, where an objective function that represents optimization criteria is minimized. System dynamics and other constraints are satisfied and the states and controls of the model such as joint kinematics and muscle recruitment patterns are optimized. The optimal control approach has demonstrated a lower computational cost in the predictive simulation of human gait using a three dimensional (3D) model^29^. When the predictive simulation includes a term in the objective function that minimizes the difference between experimental and estimated kinematics and GRF, it is also referred to as tracking simulation. To date, predictive simulation methods have been used to investigate pathologic gait patterns in cerebral palsy^30^ as well as in post-stroke^31^ and crutch-assisted walking^32^. However, few studies using complex 3D muscle-driven models compared predictions to experimental pathologic gait data and the clinical application of these methods still presents challenges such as inter-individual variability^33,34^, which is greater in stroke patients than in healthy individuals^10^.

We have observed different gait patterns and responses to FES among stroke patients with drop foot pathology in our gait laboratory. The FES device provides us with an opportunity to characterize gait in our patients with the device deactivated or activated^22^. Both gait conditions were analyzed using ID for a patient presenting with considerable gait disturbance. Tracking simulation, in which there is a trade-off between predicted and prescribed gait patterns, was performed in order to determine how the predictive model formulation affects the observed pathologic gait pattern compared to ID by solving an optimal control problem using a published model^29^. A predictive simulation of a healthy gait pattern was generated using a similar approach to estimate how the subject would walk without impairment. In order to predict impaired gait, a parameter estimation was performed whereby altered muscle–tendon parameters were calculated based on observed joint moments^30^. The predictive simulation of the impaired gait represented gait abnormalities without the use of experimental kinematics and kinetics. This allowed us to perform exploratory cause-effect analyses for the pathologic gait and compare the results of the different methods and conditions. The objectives of this work were as follows: (1) to explore the main differences between ID, tracking, and predictive simulation results of a post-stroke patient; (2) to investigate the possible causes of three gait abnormalities presented by the subject (i.e., knee hyperextension, SKG, and drop foot); and (3) to evaluate the effects of FES on the gait of the patient.

## Methods

### Experimental data

We investigated a post-stroke patient (female; age, 47.2 years; height, 1.60 m; body weight, 63.8 kg) treated with the ActiGait FES device. The patient suffered a left hemispheric hemorrhagic stroke 10.3 years prior to, and received FES treatment approximately 1 year prior to the measurements. The study was approved by the local Ethics Committee at Hannover Medical School (MHH) under reference no. 2489 and the patient provided written and informed consent to participate. All methods were performed in accordance with relevant guidelines and regulations. Overground gait was performed at a self-selected gait speed. Motion capture data were obtained with an optical infrared system consisting of 12 MX cameras controlled by Nexus v1.8.5 software (Vicon Motion System, Oxford, UK) at a sampling rate of 200 Hz and with the retroreflective markers attached to the subject in accordance with the Plug-in Gait model^35^. GRF was measured at 1 kHz using two force plates (Type BP400600; AMTI, Watertown, MA, USA). Two conditions of the patient were recorded in the same session: the unassisted drop foot (DF) condition and the FES condition using the ActiGait device on the right (ipsilateral) paretic side of the patient. The pulse timing and duration of the device was adjusted to the patient in order to obtain functional movement of the ankle and a physiologic gait pattern (standard stimulation parameters 1.1 mA, 20–30 Hz; impulse duration of 70 µs)^21,22^. The preferred gait speed in the DF and FES conditions were 0.55 ± 0.04 and 0.95 ± 0.05 m/s, respectively. A static trial used to scale the model was recorded and four gait trials for each condition were processed.

Inverse kinematics, ID, kinematics analysis, and static optimization were performed using the default tools in OpenSim v3.3^36^ to obtain joint angles, moments and velocities, and muscle forces. The 3D lower body model gait2392 was used and the metatarsophalangeal joint was locked. Model scaling was performed in OpenSim using the static trial. The results obtained in OpenSim for each condition are referred to as ID results in the comparisons (ID-DF and ID-FES).

### Optimal control problem formulation

Parameter estimation, tracking, and predictive simulation were formulated as an optimal control problem. The frameworks used in this study were developed by Falisse et al.^29,30^. The optimal control problem was transcribed into a nonlinear programming problem using CasADi^37^ and the resultant optimization problem was solved using IPOPT^38^. Both software were implemented in MATLAB (R2019a). Direct collocation with a third-order Radau collocation scheme was used for transcription^29^.

The tracking simulation included terms in the objective function that minimized the difference between the optimized variables and ID results. The objective function $J_{Track}$ was defined as follows:

1 $$J_{{Track}} = \int\limits_{{ti}}^{{tf}} {\sum {\left( {W_{{T1}} \left( {q - q_{R} } \right)^{2} + W_{{T2}} \left( {Mj - Mj_{R} } \right)^{2} + W_{{T3}} \left( {Fr - Fr_{R} } \right)^{2} + W_{{T4}} \left( {Mr - Mr_{R} } \right)^{2} + W_{{T5}} a^{2} + W_{{T6}} ( \ddot{q}^{2} + \dot{a}^{2} + \dot {Fm}^{2} )} \right)} } dt,$$

where $ti$ and $tf$ are the initial and final times, respectively; $q$ and $Mj$ are the joint angle and moment, respectively; $Fr$ and $Mr$ are the ground reaction force and moment, respectively; the subscript $R$ represents the experimental data; $a$ and $Fm$ are the muscle activation and tendon force, respectively; and $W_{T1 - 6}$ are the weight factors.

Each trial of the ID was separately tracked and the weight factors varied between trials (Supplementary Table S3). The body model (21 degrees of freedom and 92 muscles) used in ID was adapted using Hill-type muscle–tendon model^39,40^ and Raasch’s model^41,42^ to describe muscle activation dynamics. Muscle–tendon lengths, velocities, and moment arms were defined as polynomial function of joint positions and velocities^29,43^. Foot–ground interaction was modeled as Hunt-Crossley contact^36,44^ using six spheres, the parameters of which were optimized during the tracking simulation. Additional details on the model can be found elsewhere^29^.

The model included a formulation of passive joint moments, representing the effect of passive structures and generating a moment in the opposite direction when the joint angle exceeded a certain limit. The net internal joint moment was calculated as the sum of the passive moment and moment generated by the muscles. An exponential function was used to represent the passive joint moment $M_{pass}$:

2 $$M_{{pass}} = K_{{pass1}} \exp \left( {K_{{pass2}} \left( {q - \theta _{{pass2}} } \right)} \right) + K_{{pass3}} \exp \left( {K_{{pass4}} \left( {q - \theta _{{pass1}} } \right)} \right) - 0.001\dot{q},$$

where $K_{pass1 - 4}$ are the stiffness parameters and $\theta_{pass1 - 2}$ are the joint angle limits. In order to investigate the effects of the passive moment generated during knee hyperextension, three sets of parameters that determine the passive knee flexion moment ($K_{pass1}$, $K_{pass2}$, and $\theta_{pass2}$) were applied in the tracking simulation for DF. In the first parameter set (PM-Def), the default values of the passive moment parameters used by Falisse et al.^29^ were applied. In a second set (PM-High), the parameters were changed to increase the passive knee flexion moment that can be attained. Finally, in a third set (PM-None), the extension angle limit was increased beyond the knee range of motion (ROM), resulting in no passive moment being generated. The specific values used are shown in Supplementary Table S1 and the resultant passive moment curves are shown in Supplementary Fig. S1. The tracking simulations of DF were performed with all three parameter sets (Track-DF_PM-Def_, Track-DF_PM-High_, and Track-DF_PM-None_), while the tracking simulation of the FES condition (Track-FES) was performed using only the PM-High set.

The formulation and musculoskeletal model used in the predictive simulation were similar to those in the tracking simulation, but the objective function did not include the tracking terms. Thus, the gait pattern derived from the simulation was independent of the experimental (kinematics, kinetics and GRF) data. Other terms were included in the objective function $J_{Pred}$:

3 $$J_{{Pred}} = \int\limits_{{ti}}^{{tf}} {\sum {\left( {W_{{P1}} a^{2} + W_{{P2}} \dot{E}^{2} + W_{{P3}} \ddot{q}^{2} + W_{{P4}} (\dot{a}^{2} + \dot{Fm}^{2} )} \right)} } \frac{1}{{Dist}}dt,$$

where $\dot{E}$ is the metabolic energy rate, $Dist$ is the distance traveled by the pelvis in the forward direction, and $W_{P1 - 4}$ are the weight factors. Solving the optimal control problem with this objective function allowed the prediction of gait. Since the weight factors have an impact on the predicted gait pattern^28,29^, the values were kept constant across all predictive simulations (Supplementary Table S3). The passive knee moment set PM-High was used. Gait speed was imposed but the stride time of the gait cycle was optimized. A complete gait cycle was simulated and periodicity was imposed.

In order to represent the healthy gait pattern, the predictive simulations of normal gait (Pred-Normal) were performed using the generic muscle–tendon parameters obtained after the static scaling in OpenSim (i.e., the same as those used in ID and tracking simulations). Two experimental and three faster gait speeds were used in the simulations: 0.55, 0.95, 1.10, 1.40, and 1.70 m/s (Pred-Normal_0.55_, Pred-Normal_0.95_, Pred-Normal_1.10_, Pred-Normal_1.40_, and Pred-Normal_1.70_, respectively).

Predictive simulation of the DF condition (Pred-DF) was performed to investigate the effect of changes in the musculoskeletal system on pathologic gait. The simulation was performed at 0.55 m/s gait speed. In this case, the generic muscle–tendon parameters were replaced with personalized ones obtained in the parameter estimation, which was formulated as an optimal control problem^30^ and the muscle redundancy problem was solved^40^. The personalized muscle–tendon parameters for the patient performing the DF gait were estimated (additional details can be found in Supplementary Information, and values are presented in Supplementary Table S2). In order to investigate the causes of the above-mentioned gait abnormalities, the maximal isometric forces of the knee flexors (KF) and extensors (KE) and the ankle dorsiflexors (AD) and plantarflexors (AP) of Pred-DF were individually decreased and increased by 50%, representing weak and strong conditions, respectively (i.e., Weak-KF, Weak-KE, Weak-AD, Weak-AP, Strong-KF, Strong-KE, Strong-AD, and Strong-AP). The gait speed of the DF condition (0.55 m/s) was imposed in these simulations. Supplementary Table S5 shows the muscles spanning the knee and ankle and major muscles depicted in the figures. In order to investigate the effects of FES, the muscle activation weight factor $W_{P1}$ of the objective function in Eq. (3) for the ankle dorsiflexor muscles was set to 1% of the value used in Pred-DF. This condition (Pred-FES) was performed at 0.95 m/s gait speed. Details of the settings used in tracking and predictive simulations can be found in the Supplementary Information. A sensitivity analysis was performed for the predictive simulations with different parameters altered (i.e., objective function weight factors, initial guess, metabolic energy model, parameters for foot–ground contact spheres, passive knee moment set, and muscle–tendon parameters [Supplementary Information]).

### Data processing

We used three metrics to evaluate gait abnormalities. For knee hyperextension, mean knee extension (MKE) angle from the initial contact to the end of the terminal stance phase of the gait cycle was calculated. For SKG, the peak of knee flexion (PKF) angle during the swing phase was calculated. The drop foot was represented by the mean ankle dorsiflexion (MAD) angle during the swing phase. Knee flexion velocity at toe-off, ankle push-off work, leg circumduction, and early and late braking impulses^45^ were also computed. Early and late braking impulses were calculated as the areas of the posterior component of GRF curve (i.e., negative anterior-posterior GRF) preceding and following the propulsion, respectively. Circumduction was calculated as the maximum lateral displacement of the calcaneus body origin (tracking and prediction results) and of the ankle marker (ID results).

Since the ID and tracking simulation results were based on kinematic and GRF data collected during gait analysis, each condition had four trials; the mean and standard deviation were calculated after normalizing the gait cycle. Each predictive simulation condition yielded a single result that was also normalized. The ID and tracking results were filtered using a low-pass second-order Butterworth filter in MATLAB. All simulations were performed on the same workstation (3.60 GHz Intel Core i3 processor). The central processing unit time, number of iterations, and stride time of the simulations are presented in Supplementary Table S4.

## Results

### Passive knee moment

**Figure 1:**
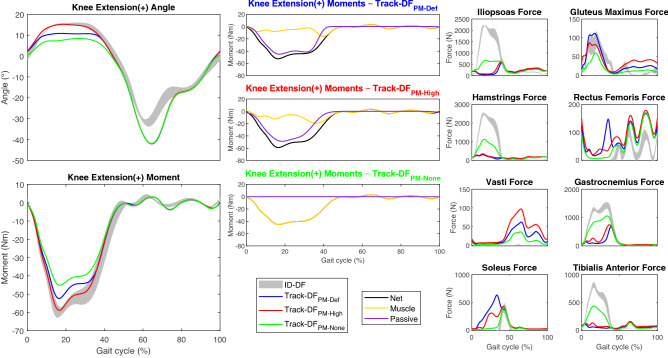
Influence of passive knee moment on the ID (mean ± standard deviation) and tracking of gait in the DF condition (ipsilateral knee angle, moments, and major muscle forces).

The passive component of the knee joint moment changed with the representation of passive moment in the DF condition (Fig. 1). Only Track-DF_PM-High_ was able to adequately represent the hyperextension and knee flexion moment of ID-DF during the stance phase. The MKE and mean knee moment during the same period in ID-DF were 12.3° and − 41.6 Nm, respectively; the values in Track-DF_PM-High_ were similar at 12.4° and − 39.8 Nm. The difference was greater in Track-DF_PM-None_ (6.4° and − 31.4 Nm) and intermediate in Track-DF_PM-Def_ (9.2° and − 35.7 Nm). As expected, no passive moment was observed in Track-DF_PM-None_ (Fig. 1 and Supplementary Fig. S1). Both ID-DF and Track-DF_PM-None_ resulted in high forces calculated for the iliopsoas, hamstrings, gastrocnemius, and tibialis anterior muscles during the stance phase (Fig. 1). There was good tracking of the ipsilateral joint angles and moments except for the PKF, peak ankle dorsiflexion, and ankle plantarflexion during the swing phase (Supplementary Fig. S2 and Video S1).

### Muscle–tendon parameters

**Figure 2:**
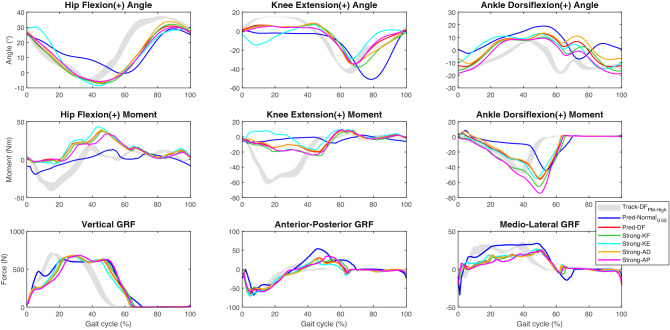
Influence of altered muscle–tendon parameters on Track-DF_PM-High_ (mean ± standard deviation), Pred-Normal_0.55_, Pred-DF, and Strong gait (ipsilateral hip, knee, and ankle angles, moments, and GRF). All simulations were performed at 0.55 m/s.

Pred-Normal_0.55_ did not reveal the abnormalities seen in Track-DF_PM-High_ and yielded a more natural gait, with the exception of hip angle, which showed reduced ROM (Fig. 2 and Supplementary Video S2). All of the joint moments were decreased except ankle dorsiflexion at the beginning of the gait cycle. On the other hand, Pred-DF showed knee hyperextension, SKG, and drop foot. MKE increased from − 2.1° in Pred-Normal_0.55_ to 5.2° in Pred-DF, while PKF decreased from 51° to 35.2° and MAD decreased from 0.04° to − 3.9°. Compared to Pred-DF, Strong-KE corrected knee hyperextension, created knee extension moment, and increased posterior GRF in the early stance phase while PKF was decreased (Fig. 2 and Supplementary Video S3). Strong-AD increased the ankle dorsiflexion angle and moment. Strong-AP and Strong-KF slightly improved knee hyperextension and SKG, respectively. The hip was most affected by Strong-KE, which increased hip ROM in the stance phase (Fig. 2). Regarding gait abnormalities, most of the effects of knee and ankle muscle weakening were the opposite of the strengthening. Weak-KF and Weak-AD decreased knee hyperextension, Weak-AP increased ankle dorsiflexion, and Weak-KE corrected the SKG, resulting in knee flexion during the swing phase similar to Pred-Normal_0.55_ (Supplementary Fig. S3 and Video S3).

**Figure 3:**
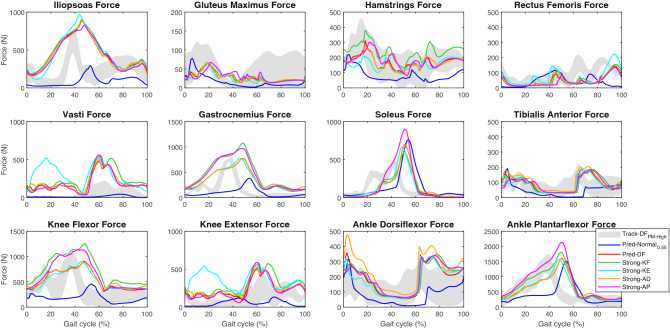
Influence of altered muscle–tendon parameters on Track-DF_PM-High_ (mean ± standard deviation), Pred-Normal_0.55_, Pred-DF, and Strong gait (ipsilateral major muscle forces). All simulations were performed at 0.55 m/s.

Pred-Normal_0.55_ showed less muscle force than Track-DF_PM-High_ for all muscles with the exception of the soleus (Fig. 3). Forces were increased in Pred-DF compared to Pred-Normal_0.55_. The sum of muscles spanning the knee and ankle joints showed that Strong-KF and Strong-AP increased the force in both muscle groups. Strong-KE also affected the hip and ankle dorsiflexor muscles; and Strong-AD had little effect on other muscles (Fig. 3). Weakening the muscle groups had the opposite effect to strengthening these muscles (Supplementary Fig. S4).

### FES

**Figure 4:**
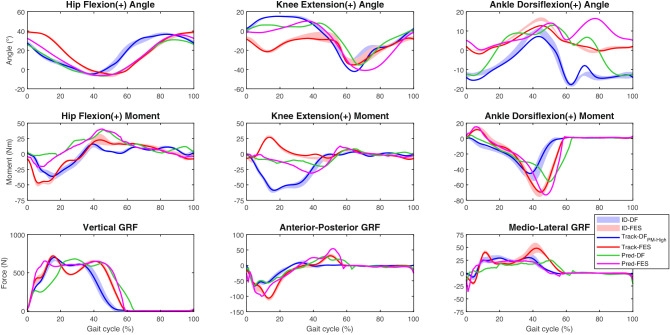
Effect of FES on ID (mean ± standard deviation), tracking, and prediction DF gait (ipsilateral hip, knee, and ankle angles, moments, and GRF). Contralateral results are presented in Supplementary Fig. S5.

In the ID results, FES corrected knee hyperextension and drop foot but did not improve SKG compared to DF (Fig. 4 and Supplementary Video S1). Thus, MKE for the ID-DF condition was 12.3° compared to − 12.1° in the ID-FES gait; PKF was 32.1° and 33.2°, respectively, and MAD increased from − 13.8° in ID-DF to 1.1° in ID-FES. Hip ROM and moment during the stance phase and ankle joint moment were increased in ID-FES. ID-DF resulted in a knee flexion moment during the stance phase while ID-FES yielded a knee extension moment. The values of GRF components were also increased in ID-FES compared to ID-DF. Peak ankle dorsiflexion was not well tracked in Track-DF_PM-High_ and Track-FES, with deviations observed between ID and tracking results (Fig. 4). No gait abnormalities were observed in the contralateral leg. FES had less effect on joint angles and moments, and both the DF and FES conditions were well tracked (Supplementary Fig. S5). The effects of FES on Pred-DF in the ipsilateral leg were similar to those of ID-FES except on the knee joint, where the hyperextension was improved only at the beginning of the cycle and the extension moment was not created in Pred-FES. Pred-DF and ID-DF showed longer and shorter stance phases, respectively, than all other results. GRF components in Pred-FES were similar to those in ID-FES except for the medio-lateral GRF (Fig. 4).

**Figure 5:**
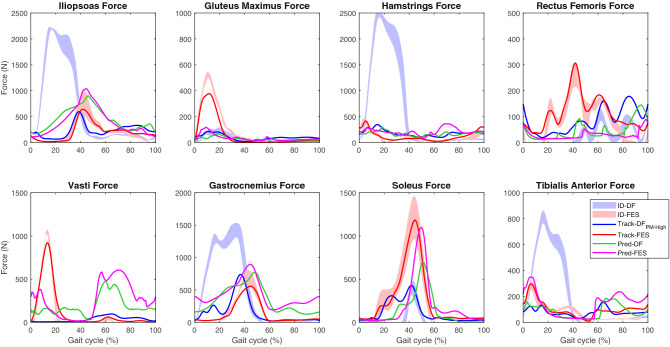
Effect of FES on ID (mean ± standard deviation), tracking, and prediction DF gait (ipsilateral major muscle forces). Contralateral results are presented in Supplementary Fig. S6.

Muscle forces were similar in Track-FES and ID-FES. FES increased gluteus maximus, rectus femoris, vasti, soleus, and tibialis anterior muscle forces compared to Track-DF_PM-High_. The effects of Pred-FES and Pred-DF on iliopsoas, soleus, and tibialis anterior muscles were similar to those of Track-FES and Track-DF_PM-High_ (Fig. 5). The forces of the contralateral muscles for ID-DF and ID-FES were similar to those for Track-DF_PM-High_ and Track-FES, respectively (Supplementary Fig. S6). The ID gait pattern of a representative trial for each condition showed that the direction of the ipsilateral resultant GRF vector moved from the anterior (in DF condition) to the posterior of the knee during the use of FES (Supplementary Fig. S7 and Video S1).

### Gait metrics

**Figure 6:**
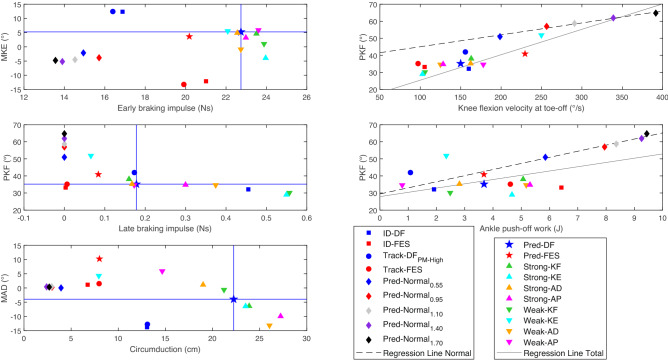
Relationship between MKE and early braking impulse; PKF and knee flexion velocity at toe-off; PKF and late braking impulse; PKF and ankle push-off work; and MAD and circumduction. Only values for the ipsilateral leg are shown. The horizontal and vertical blue lines indicate the values for Pred-DF. The dashed regression line was calculated based on the five Pred-Normal results, while the solid regression line was calculated based on all results.

The increased speed in Pred-Normal resulted in increased PKF, knee flexion velocity at the toe-off, and ankle push-off work, while MKE and MAD were less affected (Fig. 6 and Supplementary Fig. S8). The knee flexion velocity and ankle push-off work were linearly related to PKF, with all values being near to the normal regression lines. FES decreased circumduction and increased the early braking impulse in both ID and tracking (Fig. 6). Weak-KE showed a high knee flexion velocity compared to the other results at the same gait speed (0.55 m/s), while ID-FES and Track-FES showed lower knee flexion velocity at the gait speed of 0.95 m/s. Weak-KE and Track-DF_PM-High_ did not show a relationship between ankle push-off work and PKF similar to other results, but ID-DF was closer to the total regression line (Fig. 6). The decreases in MKE and PKF in the Pred-DF alterations were related to increases in early and late braking impulses, respectively, except for Pred-FES and Weak-AD. Weak-KF, Weak-KE, Weak-AP, Strong-AD, and Pred-FES increased MAD and decreased the circumduction of Pred-DF as in the FES condition compared to DF for tracking and ID (Fig. 6). Different settings yielded similar results for MKE, PKF, and MAD in the predictive simulations (Supplementary Fig. S9).

## Discussion

In this work, we compared the gait of a post-stroke patient under different conditions using ID, tracking, and predictive simulations. The ID and tracking of the ipsilateral knee angle, moments, and muscle forces showed greater deviation in DF than in the FES condition. The predictive simulation of the post-stroke patient represented gait abnormalities as well as a normal gait at different gait speeds; changing the maximal isometric force of muscle groups spanning the knee and ankle corrected the predicted gait abnormalities. Additionally, predictive simulation of the effect of FES corrected the drop foot, but there were deviations from the ID and tracking results.

The patient we investigated presented with knee hyperextension, where the passive structures posterior to the knee play a greater role in controlling further extension^3,13^. In tracking the DF condition, we observed that the passive knee moment affected the ability of the model to track ID knee hyperextension and knee flexion moment in the stance phase. The knee flexion moment pattern is related to knee hyperextension^12^. If the passive moment was not represented in the model such as in ID, the knee flexion moment in the hyperextended knee is generated entirely by the knee flexor muscle force (i.e., hamstrings and gastrocnemius muscles), misrepresenting the actual situation. Both muscles are biarticular and also act as hip extensor and ankle plantarflexor, respectively. These forces are in turn counterbalanced by the antagonist muscles to achieve the hip and ankle moments. Consequently, the forces of the iliopsoas and tibialis anterior muscles, which are the above-mentioned antagonist muscles (i.e., hip flexor and ankle dorsiflexor muscles, respectively), were also misrepresented (Fig. 1). This problem was apparent in our subject, who presented with drop foot pathology: the ID increased the tibialis anterior muscle force during drop foot gait, which the patient could not generate and with no net ankle dorsiflexion moment produced. This was also illustrated by Track-DF_PM-None_ in which the net knee flexion moment was created solely by the muscles, as the onset of the passive knee flexion moment would only occur at 22° of extension, which was not reached by the subject (Supplementary Fig. S1). This caused a similar result to that observed in ID-DF. For Track-DF_PM-Def_ and Track-DF_PM-High_, the net knee flexion moment was mainly generated by the passive moment. Consequently, less muscle force was predicted for the hamstrings, gastrocnemius, iliopsoas, and tibialis anterior muscles. As Track-DF_PM-High_ created more passive moment than Track-DF_PM-Def_ (Supplementary Fig. S1), the peaks of the knee hyperextension and knee flexion moment increased to the values observed in ID-DF. During FES, where the passive moment was not generated, similar muscle forces were observed with both methods (ID-FES and Track-FES). The same was observed on the contralateral side for both conditions. Thus, representing the passive flexion moment caused by passive dorsal structures of the knee during hyperextension is important in order to prevent the calculation of non-physiologic muscle forces. This also illustrates the importance of representing the passive moment when estimating muscle force^46,47^, especially in gait patterns where the joint ROM differs significantly from the healthy condition as is the case in knee hyperextension.

During healthy gait at a natural cadence, the knee joint is flexed during the early stance phase and the peak extension moment is generated by the quadriceps muscles^13,48^. The increases in ipsilateral knee flexion angle and knee extension moment in ID-FES, Track-FES, Strong-KE, Weak-KF, and Weak-AD during the early stance were accompanied by an increase in vasti muscle force, while the ID-DF, Track-DF_PM-High_, and Weak-KE resulted in less force. This suggests a possible association between knee hyperextension and knee extensor weakness in our stroke patient^13,49^. The same was also observed on the contralateral side for ID, tracking and prediction of FES and DF, which presented knee flexion and vasti muscle force in early stance. The lack of vasti muscle force could also explain why Pred-Normal showed low knee flexion during the stance phase. Although Pred-DF showed more vasti muscle force than Pred-Normal_0.55_, the personalized muscle–tendon parameters altered the balance among several muscles, causing knee hyperextension and other gait abnormalities.

Knee hyperextension in mid-stance is often attributed to ankle plantarflexor weakness, especially as the gastrocnemius muscle physiologically produces a knee flexion moment during this phase^3,11^. In this study, hyperextension was increased slightly in the stance phase in Weak-AP and was decreased in the early and late stance but not during mid-stance phase in Strong-AP, which is not consistent with the literature^3^. Like Strong-AP, Strong-KF reduced hyperextension but to a lesser extent. Both simulations resulted in an increased knee flexion moment; however, since the knee flexor muscles were able to generate more force, less passive moment was needed compared to Pred-DF and hyperextension was reduced.

A GRF vector anterior to the knee creates an external moment that extends the knee and may cause knee hyperextension^11,50^. This also occurred during the initial contact in the DF condition, as the center of pressure (origin of the GRF) was shifted distally from the heel (i.e. more anteriorly) due to the increased plantarflexion. During FES, the foot was placed on the ground in a dorsiflexed position, with the center of pressure shifted back to the heel. As a result, the experimental GRF was directed posteriorly to the knee, allowing for correction of the knee hyperextension (Supplementary Fig. S7 and Video S1). A similar outcome was reported in a case study^19^. A related parameter, the braking impulse, is defined as the time integral of the posterior component of GRF and indicates the degree to which braking is present during stance. The early braking impulse was increased in ID-FES and Track-FES compared to ID-DF and Track-DF_PM-High_, as well as in Strong-KE and Weak-KF, which clearly reduced hyperextension compared to Pred-DF (Fig. 6). An increase in braking impulse in severe stroke patients has been linked to increased vastus lateralis muscle force in the early stance phase^51^. This is consistent with our observation that increased vasti muscle force in Strong-KE and Weak-KF was related to the increase in early braking impulse compared to Pred-DF. The same relationship was observed in the comparison between FES and DF conditions for tracking and ID, but not in Pred-FES and Weak-AD compared to Pred-DF.

Knee hyperextension has also been attributed to early activity of the calf muscles and forefoot landing^10,12^. ID-FES and Track-FES delayed the onset of gastrocnemius muscle force and improved ankle kinematics during the initial contact phase on the ipsilateral side. This response was not obvious in the predictive simulation when hyperextension was decreased, as Weak-AD presented reduced ankle dorsiflexion at initial contact and reduced knee hyperextension compared to Pred-DF.

Track-DF_PM-High_ showed less ankle push-off work and more knee flexion in the swing phase than ID-DF whereas for FES, the difference between the two methods was more apparent in the ankle push-off, where a decrease was also observed. It is unclear why the ipsilateral PKF was not well tracked only in the DF condition, which showed impaired ankle push-off work that could explain SKG^2^. Pred-FES did not improve the push-off work compared to Pred-DF. FES improved the push-off in tracking and ID, as reported by others^52^, but SKG was not improved. This may be attributable to the increased action of the ipsilateral rectus femoris muscle in the stance phase (Fig. 5), as hyperreflexia or overactivity of the rectus femoris muscle are among the main causes of SKG^14^. In ID-FES and Track-FES, the contralateral rectus femoris muscle force was delayed relative to the ipsilateral side. Interestingly, it was suggested that rectus femoris muscle spasticity can be triggered following an improved push-off and gait speed^2^. Indeed, the increase in these gait parameters was accompanied by increased rectus femoris muscle action in ID-FES and Track-FES compared to ID-DF and Track-DF_PM-High_, but the spasticity was not examined in this work. Clarifying the causes of SKG could improve the efficacy of botulinum toxin injection into the rectus femoris muscle. In our patient, the treatment may be more effective during FES, because impaired push-off could be the main cause of SKG in the DF condition.

The changes in maximal isometric force of the knee muscles affected SKG in Pred-DF. Both Weak-KF and Strong-KE decreased knee flexion in the swing phase. The opposite effect was observed in Strong-KF and Weak-KE, suggesting that knee flexor weakening may cause SKG^10^. We observed an association between SKG and increased late braking impulse, but SKG was related to decreased ankle push-off work only in Strong-KF and Weak-KF (Fig. 6). Both mechanisms were shown to affect knee flexion during the swing phase^2,45^. Strong-AP and Weak-AD increased the ankle push-off work of Pred-DF, whereas the opposite was true in Weak-AP and Strong-AD. This indicates that the weakness of the ankle plantarflexor muscles may be related to impaired ankle push-off, since these muscles contribute to this mechanism^53^. However, changes in the strength of these muscle groups had little effect on PKF.

In previous simulations, increased iliopsoas and gastrocnemius muscle forces increased knee flexion velocity, which was decreased by higher rectus femoris, vasti and soleus muscle forces^54^. Knee flexion velocity at toe-off in Weak-KE, which decreased vasti and rectus femoris muscle forces, was increased to a value close to Pred-Normal_0.95_. This could explain the correction of SKG in this result, which showed a knee flexion pattern similar to Pred-Normal_0.55_ during the swing phase. The opposite effect was observed in Strong-KE. Weak-KF and Strong-KF affected the gastrocnemius muscle force to a greater extent than the soleus muscle force, decreasing and increasing, respectively, the knee flexion velocity, as well as PKF. Thus, the changes in the strength of knee muscles indicated an association between knee flexion velocity at toe-off and PKF, which has been reported by other studies^15,55^. However, in ankle muscles, the change in knee flexion velocity had little effect on PKF. Although gait speed in the FES condition was nearly two times higher than in the DF condition, knee flexion velocity at toe-off was decreased in ID and tracking, possibly due to the action of knee extensor muscles that enhanced the braking mechanism^2^. Pred-FES showed a higher knee flexion velocity and PKF than Pred-DF.

Weak-AD increased drop foot compared to Pred-DF while the strengthening of these muscles increased ankle dorsiflexion during the swing phase, implying that weak ankle dorsiflexors is a potential cause of the drop foot^1^. Weak-AP increased MAD, supporting the possibility that spasticity of these muscles prevents ankle dorsiflexion^56^.

Gait under FES showed increased ankle dorsiflexion compared to the DF condition, which is the desired outcome of this treatment^19,52^. The lack of dorsiflexion in the late swing phase of stroke patients may affect the ankle during initial contact, causing a flat foot or even a forefoot landing and decreasing ankle dorsiflexion moment^16,56^, which was observed in our study when drop foot increased. Drop foot patients may also present circumduction of the leg^21^, which is a potential strategy to achieve foot clearance^10,12^. In our study, the drop foot correction was accompanied by decreased circumduction in the different conditions of the ID, tracking, and Pred-DF results, while decreased ankle dorsiflexion in the swing phase was accompanied by increased circumduction (Fig. 6). However, the circumduction was unrelated to the late braking impulse in our results, which is in disagreement with previous work demonstrating a link between a large late braking impulse and increased circumduction^45^. It is interesting to note that ID-DF and Track-DF_PM-High_ presented the same value for ipsilateral circumduction and that Pred-FES, ID-FES, and Track-FES values were similar, with Pred-DF showing more circumduction.

Pred-Normal results were similar to those of healthy gait, which confirmed that the gait abnormalities present in Pred-DF were caused by altered muscle–tendon parameters, as that was the only difference between these results. The effect of increased gait speed in Pred-Normal is supported by earlier reports^24,25^, except for the lack of knee flexion during the stance phase at faster speeds, which has also been reported in other predictive simulation studies^28,29^. The different gait speeds showed a linear relationship between the increases in PKF, ankle push-off work, and knee flexion velocity, which has also been described by others^2^.

The developers of the framework used in our work validated that the model could predict the relationship between gait speed and metabolic cost of transport during healthy gait^29,34^. Using a similar model, personalized muscle–tendon parameters allowed the prediction of the crouch gait pattern of a child with cerebral palsy^30^. Nonetheless, the validation of predictive simulations is, in general, still limited^33,34^.

A limitation of the present work was the use of a generic musculoskeletal model that did not include subject-specific characteristics. As neither magnetic resonance imaging (MRI) nor electromyography (EMG) data were collected, it was not possible to create a personalized musculoskeletal model. Ultrasound imaging, MRI and EMG data could allow validation and improve the personalization of muscle–tendon parameters, particularly where the values may not accurately represent the physiology and anatomy of the patient. Another limitation is that we analyzed only one patient, since the focus of this study was on the application of the methodology. Therefore, the results and conclusions may not be generalized to the whole stroke population. Additionally, this work could be improved by more accurate modeling of FES in the predictive model. Future studies on pathologic gait using these methods in more patients could lead to their application in a clinical context. For instance, predictive simulation could be used to evaluate how the gait of stroke patients is affected by different treatment options such as use of orthoses, FES, and botulinum toxin injection. This would allow individualized therapeutic recommendations or even recommendations based on level of motor impairment, time since stroke, and age.

A limitation of the optimal control problem is the lack of confidence that the results represent a global optimum. To address this issue, all predictive simulation results were solved using a different initial guess for joint kinematics. The weight factors of objective functions of parameter estimation, tracking, and predictive simulation were manually tuned. In the tracking simulation, the values that yielded the gait pattern most similar to the experimental data were selected for each trial. The different values of weight factors had no impact on the knee hyperextension observed during the tracking simulation. Parameter estimation and predictive simulations were performed using different sets of weight factors. Other settings were altered in the predictive simulation during the sensitivity analysis; simulations that converged for all conditions and predicted a human-like gait pattern were analyzed. Although differences were observed in some results, most of the gait patterns were similar to those presented in this work and the main conclusions were the same.

## Conclusion

This study investigated the causes of abnormalities and improvements in the gait under different conditions of a stroke patient using ID, tracking, and predictive simulations. The results showed that the passive knee moment plays an important role in gait when joint ROM is beyond the physiologic values as in patients with knee hyperextension. Tracking simulation reproduced the gait patterns observed in ID. Predictive simulation of healthy gait at different speeds captured relationships reported in the literature. Altered muscle–tendon parameters allowed us to predict gait abnormalities, but differences were observed in comparison to the experimental results. Increase of knee extensor strength predicted a reduction in knee hyperextension. SKG was corrected by the weakening of the knee extensors and strengthening of the knee flexors. Weak ankle plantarflexors and strong ankle dorsiflexors predicted a reduced drop foot. During the use of FES, we observed a corrected knee hyperextension and drop foot, whereas SKG was not improved. It is possible that the increased activity of the rectus femoris limited the peak of knee flexion in swing phase during this condition. FES also improved gait speed and reduced circumduction, which was predicted in our work. Therefore, we demonstrated that these methods have the potential to aid the investigation of the causes of gait impairments and the design of individualized rehabilitation and treatment programs of post-stroke patients.

## Supplementary Information

Supplementary Information 1.

Supplementary Video 1.

Supplementary Video 2.

Supplementary Video 3.

## Data Availability

The datasets generated and analyzed during the current study are available from the corresponding author on reasonable request.
