# Supplementary material for: Predictive simulation of post-stroke gait with functional electrical stimulation
Source: Sci Rep. 2021 Nov 1;11:21351. doi: 10.1038/s41598-021-00658-z (PMC8560756; doi:10.1038/s41598-021-00658-z)
Supplement: Supplementary file 1 — Supplementary Information 1. [file 41598_2021_658_MOESM1_ESM.pdf]

Supplementary Information: Predictive simulation of post-stroke gait with functional electrical stimulation

Gilmar F. Santos[1*], Eike Jakubowitz[1], Nicolas Pronost[2], Thomas Bonis[2], and Christof Hurschler[1]

[1]Laboratory for Biomechanics and Biomaterials, Department of Orthopedics, Hannover Medical School, Hannover, Germany
[2]CNRS LIRIS, Université Claude Bernard Lyon 1, Université de Lyon, Lyon, France
[*]FernandesdosSantos.Gilmar@mh-hannover.de

ABSTRACT

Post-stroke patients present various gait abnormalities such as drop foot, stiff-knee gait (SKG), and knee hyperextension. Functional electrical stimulation (FES) improves drop foot gait although the mechanistic basis for this effect is not well understood. To answer this question, we evaluated the gait of a post-stroke patient walking with and without FES by inverse dynamics analysis and compared the results to an optimal control framework. The effect of FES and cause-effect relationship of changes in knee and ankle muscle strength were investigated; personalized muscle–tendon parameters allowed the prediction of pathologic gait. We also predicted healthy gait patterns at different speeds to simulate the subject walking without impairment. The passive moment of the knee played an important role in the estimation of muscle force with knee hyperextension, which was decreased during FES and knee extensor strengthening. Weakening the knee extensors and strengthening the flexors improved SKG. During FES, weak ankle plantarflexors and strong ankle dorsiflexors resulted in increased ankle dorsiflexion, which reduced drop foot. FES also improved gait speed and reduced circumduction. These findings provide insight into compensatory strategies adopted by post-stroke patients that can guide the design of individualized rehabilitation and treatment programs.

Passive knee moment

The passive joint moment was defined as follows:

$$M_{pass} = K_{pass1} exp(K_{pass2}(q - \theta_{pass2})) + K_{pass3} exp(K_{pass4}(q - \theta_{pass1})) - 0.001\dot{q}, \tag{S1}$$

where $K_{pass1-4}$ are the stiffness parameters, $\theta_{pass1-2}$ are the joint angle limits, and q is the joint angle.

The passive knee moment parameters used in the different sets are presented in Table S1. Figure S1 shows the relationship between the passive knee moment and sagittal angle. Knee sagittal angles between −150° and 50° were applied in the passive knee moment function in Eq. S1 and passive knee moment was calculated for the three sets. The relationship between passive knee moment and sagittal angle obtained during tracking simulations in the drop foot (DF) condition is also shown in Fig. S1.

Parameter estimation

In the parameter estimation, the personalized muscle–tendon parameters were estimated using an optimal control problem[1]. The following generic muscle–tendon parameters were obtained after scaling in inverse dynamics (ID): optimal fiber length, maximal isometric force, tendon slack length, optimal pennation angle, and maximal fiber contraction velocity (10 times the optimal fiber length).The maximal isometric force, optimal fiber length, and tendon slack length were optimized. The generic parameters used in the ID were the initial values and the muscle redundancy problem was solved[2]. The joint moments of the drop foot gait from ID (ID-DF) were reproduced. Ipsilateral knee moment was replaced with the moment obtained in Track-DF$_{\text{PM-None}}$ in order to reduce the influence of the passive moment, which was not included in the parameter estimation. The effect of knee hyperextension on muscle activation was thus reduced; moreover, it was assumed that the muscles could generate the knee moment as the tracking simulation used the same muscle model and Track-DF$_{\text{PM-None}}$ did not generate passive moment. Muscle excitation was optimized and the objective function was minimized[1]. The following objective function J_{Estim} was used:

$$J_{Estim} = \int_{ti}^{tf} \sum \left(W_{E1} a^2 + W_{E2} Re^2 + W_{E3} (\dot{a}^2 + \dot{Fm}^2) \right) dt, \tag{S2}$$

where ti and tf are initial and final times, respectively; a and Fm are muscle activation and tendon force, respectively; Re is the reserve actuator; and W_{E1-3} are weight factors (W_{E1} = 0.00750, W_{E2} = 0.99240, and W_{E3} = 0.00005).

The muscle–tendon parameters were bound between 50% and 200% of the initial values. The number of mesh intervals was 100 and the tolerance was 10^{-4}. Parameters for all muscles with the exception of those spanning the lumbar joint were estimated. The parameter estimation converged in 5400 iterations after 19.95 h of central processing unit (CPU) time. Generic and personalized muscle–tendon parameters resulting from the parameter estimation are presented in Table S2.

Tracking and predictive simulations

Each tracking simulation result presented in this work comprised four simulations, i.e., one for each trial performed during the gait analysis. The initial guess, bounds, and scaling of joint kinematics were based on the gait trial that was tracked. Details of the calculation of these parameters have been previously published[3]. Thus, the gait speed and stride time were the same as in the ID result. The number of mesh intervals in the optimization was 100 and the tolerance was 10^{-4}. The objective function for tracking was as follows:

$$J_{Track} = \int_{ti}^{tf} \sum \left(W_{T1} (q - q_R)^2 + W_{T2} (Mj - Mj_R)^2 + W_{T3} (Fr - Fr_R)^2 + W_{T4} (Mr - Mr_R)^2 + W_{T5} a^2 + W_{T6} (\ddot{q}^2 + \dot{a}^2 + \dot{Fm}^2) \right) dt, \tag{S3}$$

where q and Mj are the joint angle and moment, respectively; Fr and Mr are the ground reaction force (GRF) and moment, respectively; the subscript R represents the experimental data; and W_{T1-6} are the weight factors.

For the predictive simulation, the initial guess for joint kinematics was based on the data of a healthy subject walking used by Falisse et al.[3], representing a normal gait. The bounds of stride time varied between 2 and 2.5 s in the simulations. The bounds and scaling for joint kinematics were calculated based on one trial in the DF condition, which allowed the model to achieve the ROM observed in the pathologic gait pattern. Details of this calculation and the description of other parameters used in the problem formulation can be found elsewhere[3]. The parameters for the contact spheres used in the GRF prediction were the mean of values optimized in Track-$DF_{\text{PM-High}}$ and Track-FES. The objective function was determined with the following equation:

$$J_{Pred} = \int_{ti}^{tf} \sum \left(W_{P1} a^2 + W_{P2} \dot{E}^2 + W_{P3} \ddot{q}^2 + W_{P4} (\dot{a}^2 + \dot{Fm}^2) \right) \frac{1}{Dist} dt, \tag{S4}$$

where $\dot{E}$ is the metabolic energy rate, $Dist$ is the distance traveled by the pelvis in the forward direction, and W_{P1-4} are the weight factors.

The metabolic energy rate used in Eq. S4 was modeled as a smooth approximation of a model developed by Bhargava et al.[3,4]. The number of mesh intervals was 400 and the tolerance was 10^{-4}. Although the large number of mesh intervals increased the computational cost, the resultant predictive simulations were more robust against changes in the settings.

The weight factors used in the tracking and predictive simulations are presented in Table S3. The resultant number of iterations, CPU time, and stride time of each simulation are presented in Table S4. The function in the sagittal plane of the major muscles and muscles spanning the knee and ankle joints is presented in Table S5.

Sensitivity analysis of predictive simulations

The settings used during the predictive simulation were individually altered, and the effects of these changes on gait parameters were analyzed.

- Setting 0: ID and tracking results

- Setting 1: Presented in the main document and described above
- Setting 2: Increased muscle activation weight factor (W_{P1} = 15000)
- Setting 3: Increased metabolic energy rate weight factor (W_{P2} = 200)
- Setting 4: Increased joint acceleration weight factor (W_{P3} = 10000)
- Setting 5: Metabolic energy model developed by Uchida et al.[3,5]
- Setting 6: Generic values for the foot–ground contact sphere parameters used by Falisse et al.[3]
- Setting 7: Initial guess of a healthy subject (female; age, 26.2 years; height, 1.65 m; body weight, 56.4 kg; gait speed, 1.16 m/s) recorded in our gait laboratory
- Setting 8: Passive knee moment set PM-None was used
- Setting 9: Different parameter estimation was performed where the optimal fiber length was minimized in the objective function in Eq. S2 and different weight factors were used.

The bound of stride time varied between the simulations. Figure S9 shows the gait abnormality metrics for all settings. All other results presented in the main document and in the Supplementary Information used Setting 1.

Supplementary videos

We created three supplementary videos of gait patterns showing the views in the sagittal plane and frontal plane. Two complete gait cycles are presented in the predictive simulations.

- Video S1: Representative trial for ID-DF, ID-FES, Track-DF$_{\text{PM-High}}$, Track-FES, and GRF vector for ID
- Video S2: Pred-DF, Pred-Normal$_{0.55}$, Pred-FES, and Pred-Normal$_{0.95}$
- Video S3: Pred-DF, Strong-KE, and Weak-KE.

Supplementary tables

Table S1. Passive knee moment parameters.

Set	K_{pass1} (Nm)	K_{pass2} (1/rad)	K_{pass3} (Nm)	K_{pass4} (1/rad)	θ_{pass1} (rad)	θ_{pass2} (rad)
PM-Def	−6.09	33.94	11.03	−11.33	−2.4	0.13
PM-High	−20	15	11.03	−11.33	−2.4	0.2051
PM-None	−6.09	33.94	11.03	−11.33	−2.4	0.4363

The passive joint moment function is presented in Eq. S1. For PM-Def, the default values of the passive moment parameters used by Falisse et al.[3] were applied. For PM-High, the parameters were changed to increase the passive knee flexion moment that can be attained. For PM-None, the extension angle limit was increased beyond the knee range of motion, resulting in no passive moment being generated.

Table S2. Maximal isometric force, optimal fiber length, and tendon slack length of the parameter estimation.

Muscle	Maximal isometric force (N)			Optimal fiber length (cm)			Tendon slack length (cm)		
	Generic	Personalized		Generic	Personalized		Generic	Personalized	
	Both	Right	Left	Both	Right	Left	Both	Right	Left
Gluteus medius 1	819	1291.82	1338.41	5.49	9.36	8.47	8.00	4.00	4.00
Gluteus medius 2	573	1089.16	1146	8.66	11.41	10.96	5.43	2.72	2.72
Gluteus medius 3	653	1306	1306	6.59	9.52	9.50	5.41	2.71	2.71
Gluteus minimus 1	270	534.17	540	6.86	5.49	3.95	1.61	3.22	3.23
Gluteus minimus 2	285	570	570	5.62	3.19	2.81	2.61	5.22	4.70
Gluteus minimus 3	323	646	646	3.81	1.91	1.91	5.12	7.10	6.43
Semimembranosus	1288	1602.61	1275.32	7.55	15.09	11.57	33.86	25.32	24.98
Semitendinosus	410	408.10	284.72	18.89	33.04	36.37	24.01	12.00	12.01
Biceps femoris long head	896	1609.24	835.66	10.28	20.55	20.55	30.73	19.27	19.91
Biceps femoris short head	804	1608	910.24	16.40	8.20	19.11	8.44	15.36	4.22
Sartorius	156	312	312	50.04	31.02	44.04	9.62	19.25	4.81
Adductor longus	627	698.65	689.64	13.26	14.79	12.18	10.57	5.28	8.10
Adductor brevis	429	284.04	290.64	12.97	9.60	10.42	1.95	3.90	3.90
Adductor magnus 1	381	212.47	237.57	8.41	4.21	10.77	5.80	7.13	3.01
Adductor magnus 2	343	174.32	180.19	11.37	8.63	13.17	11.27	12.01	8.47
Adductor magnus 3	488	244.02	255.41	12.32	10.67	24.64	23.42	25.33	12.20
Tensor fasciae latae	233	466	466	9.18	5.46	10.45	41.05	43.36	36.94
Pectineus	266	278.02	197.05	9.77	8.18	4.89	3.22	1.61	4.88
Gracilis	162	81.01	81.01	33.01	18.74	20.11	11.82	23.62	23.55
Gluteus maximus 1	573	525.20	1116.03	14.41	16.73	14.76	12.69	6.35	6.34
Gluteus maximus 2	819	629.65	1272.57	14.74	17.97	15.75	12.74	6.37	6.37
Gluteus maximus 3	552	284.22	300.87	14.45	20.34	21.93	14.55	7.98	7.28
Iliacus	1073	1588.04	1194.37	10.19	5.91	16.01	10.19	12.29	5.10
Psoas major	1113	1740.58	1253.16	10.27	16.66	18.67	16.43	8.21	8.21
Quadratus femoris	381	315.64	505.66	5.48	5.19	4.94	2.43	1.22	1.68
Gemellus	164	233.92	289.20	2.40	4.17	4.36	3.90	1.95	1.96
Piriformis	444	744.46	792.01	2.67	1.34	5.34	11.81	12.53	8.75
Rectus femoris	1169	1311.47	1298.36	10.95	19.62	21.90	29.78	20.98	15.43
Vastus medialis	1294	1189.47	1195.35	8.48	4.88	11.99	12.01	12.57	6.00
Vastus intermedius	1365	1254.75	1268.96	8.30	4.78	12.96	12.97	13.50	6.49
Vastus lateralis	1871	1793.71	1805.34	8.00	5.20	12.85	14.96	14.82	7.48
Medial gastrocnemius	1558	2022.02	1455.99	5.61	11.22	2.81	36.47	29.71	37.55
Lateral gastrocnemius	683	1055.26	579.81	5.98	5.44	2.99	35.53	33.76	36.94
Soleus	3549	3257.94	3582.11	4.67	3.13	9.34	23.36	24.91	19.20
Tibialis posterior	1588	1521.44	1634.63	2.90	5.80	5.80	29.02	26.39	26.36
Flexor digitorum longus	310	237.83	292.96	3.20	1.60	1.60	37.64	37.69	38.45
Flexor hallucis longus	322	175.36	259.73	4.05	8.07	2.02	35.77	31.84	36.82
Tibialis anterior	905	1588.79	1334.94	9.19	17.31	5.28	20.91	10.45	21.78
Peroneus brevis	435	418.03	461.76	4.69	9.36	2.34	15.10	11.68	17.00
Peroneus longus	943	899.21	967.71	4.60	9.20	9.20	32.40	29.13	28.20
Peroneus tertius	180	359.99	359.99	7.40	11.73	3.71	9.37	4.69	12.31
Extensor digitorum longus	512	938.14	952.56	9.59	5.47	19.15	32.43	35.19	21.27
Extensor hallucis longus	162	324	324	10.44	5.22	5.22	28.69	32.40	31.84
Erector spinae	2500	2500	2500	11.63	11.63	11.63	2.91	2.91	2.91
Internal oblique	900	900	900	9.77	9.77	9.77	9.77	9.77	9.77
External oblique	900	900	900	11.91	11.91	11.91	13.90	13.90	13.90

Table S3. Weight factors of the objective functions for tracking and predictive simulations.

Simulation	Weight factor		Value		
Tracking	Joint angle	W_{T1}	10	20	10
	Joint moment	W_{T2}	10	10	10
	GRF	W_{T3}	5	10	15
	Ground reaction moment	W_{T4}	1	1	1
	Muscle activation	W_{T5}	1	1	1
	Joint acceleration and muscle time derivatives	W_{T6}	0.001	0.001	0.001
Predictive	Muscle activation	W_{P1}		10000	
	Metabolic energy rate	W_{P2}		100	
	Joint acceleration	W_{P3}		5000	
	Muscle time derivatives	W_{P4}		0.001	

The objective functions are presented in Eqs. S3 (tracking) and S4 (predictive). For the tracking simulations, we used the values of weight factor presented in the second column in one trial of Track-FES. The values presented in the third column were used in one trial of Track-$DF_{PM\text{-}High}$, in one trial of Track-$DF_{PM\text{-}None}$, and in two trials of Track-$DF_{PM\text{-}Def}$. The remaining 11 tracking simulations were performed using the values presented in the first column.

Table S4. Number of iterations, CPU time, and stride time for the simulations.

Simulation	Number of iterations	CPU time (h)	Stride time (s)
Track-$DF_{PM\text{-}Def}$	802 ± 289.91	5.04 ± 2.33	1.66 ± 0.11
Track-$DF_{PM\text{-}High}$	854.75 ± 364.16	4.71 ± 2.85	
Track-$DF_{PM\text{-}None}$	714.25 ± 264.12	3.73 ± 1.57	
Track-FES	548.25 ± 91.59	2.48 ± 0.48	1.19 ± 0.05
Pred-$Normal_{0.55}$	635	2.34	1.50
Pred-$Normal_{0.95}$	576	2.09	1.23
Pred-$Normal_{1.10}$	648	2.36	1.12
Pred-$Normal_{1.40}$	882	3.22	0.97
Pred-$Normal_{1.70}$	868	3.22	0.87
Pred-DF	744	2.72	1.91
Strong-KF	912	3.30	1.93
Strong-KE	932	3.39	1.98
Strong-AD	831	3.00	1.91
Strong-AP	893	3.23	1.94
Weak-KF	836	3.04	1.94
Weak-KE	643	2.35	1.82
Weak-AD	870	3.14	1.94
Weak-AP	1010	3.71	1.84
Pred-FES	852	3.15	1.25

Table S5. Function in the sagittal plane of major muscles and other muscles spanning the knee and ankle joints.

**Major muscle**	**Individual muscle**	**Function sagittal plane**	
Iliopsoas	Iliacus	Hip flexion	
	Psoas major		
Gluteus maximus	Gluteus maximus 1	Hip extension	
	Gluteus maximus 2		
	Gluteus maximus 3		
Hamstrings	Semimembranosus	Hip extension	Knee flexion
	Semitendinosus		
	Biceps femoris long head		
	Biceps femoris short head	Knee flexion	
Rectus femoris	Rectus femoris	Hip flexion	Knee extension
Vasti	Vastus medialis	Knee extension	
	Vastus intermedius		
	Vastus lateralis		
Gastrocnemius	Medial gastrocnemius	Knee flexion	Ankle plantarflexion
	Lateral gastrocnemius		
Soleus	Soleus	Ankle plantarflexion	
Tibialis anterior	Tibialis anterior	Ankle dorsiflexion	
Not presented	Gracilis	Hip flexion	Knee flexion
	Sartorius		
	Peroneus brevis	Ankle plantarflexion	
	Peroneus longus		
	Tibialis posterior		
	Flexor digitorum longus		
	Flexor hallucis longus		
	Extensor digitorum longus	Ankle dorsiflexion	
	Extensor hallucis longus		
	Peroneus tertius		

The major muscle force is the sum of individual muscle forces. The forces of the muscles spanning the knee and ankle joints that are not presented are included in the sum of the knee flexor, ankle plantarflexor, and ankle dorsiflexor forces depicted in the figures.

Supplementary figures

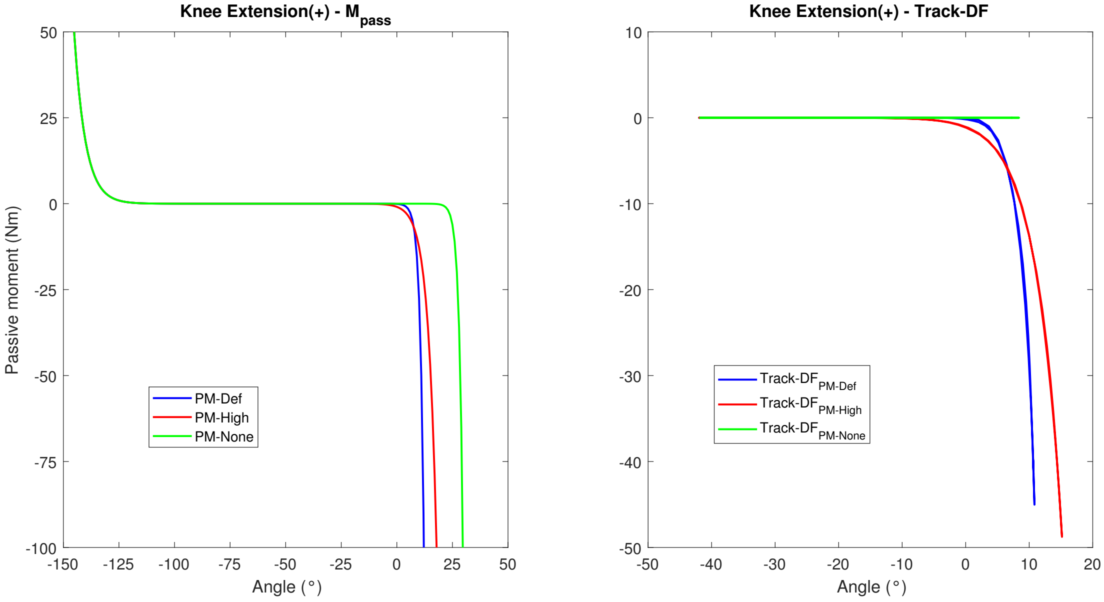

Figure S1. Relationship between passive knee moment and angle for the function in Eq. S1 and for the tracking of gait in the DF condition (ipsilateral knee joint).

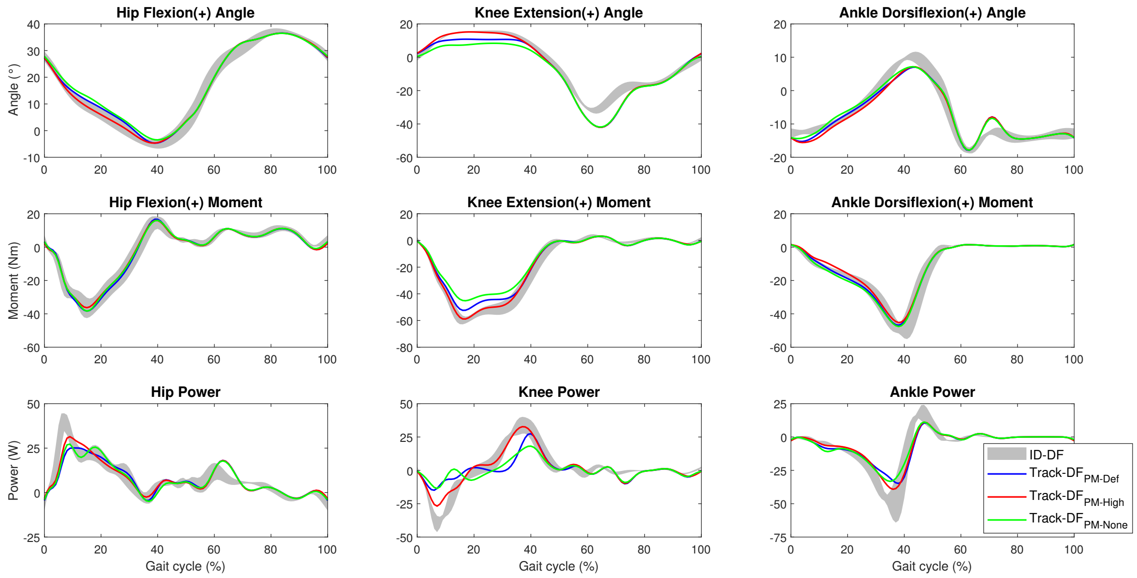

Figure S2. Influence of passive knee moment on the ID (mean $\pm$ standard deviation) and tracking of gait in the DF condition (ipsilateral hip, knee, and ankle angles, moments and powers).

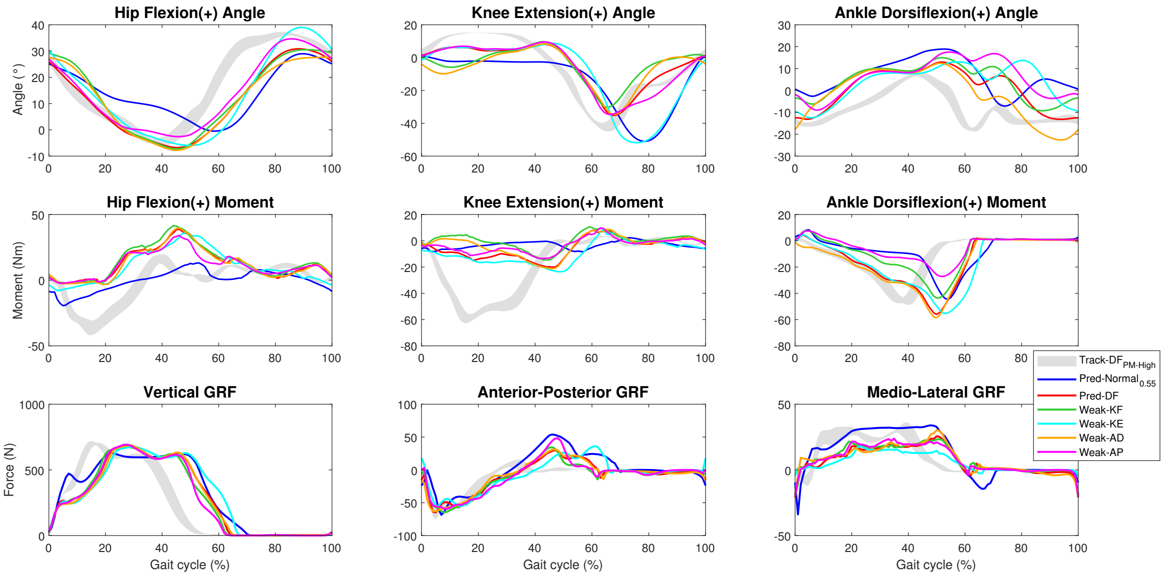

Figure S3. Influence of altered muscle–tendon parameters on Track-DF$_{\text{PM-High}}$ (mean ± standard deviation), Pred-Normal$_{0.55}$, Pred-DF, and Weak gait (ipsilateral hip, knee, and ankle angles, moments, and GRF). All simulations were performed at 0.55 m/s.

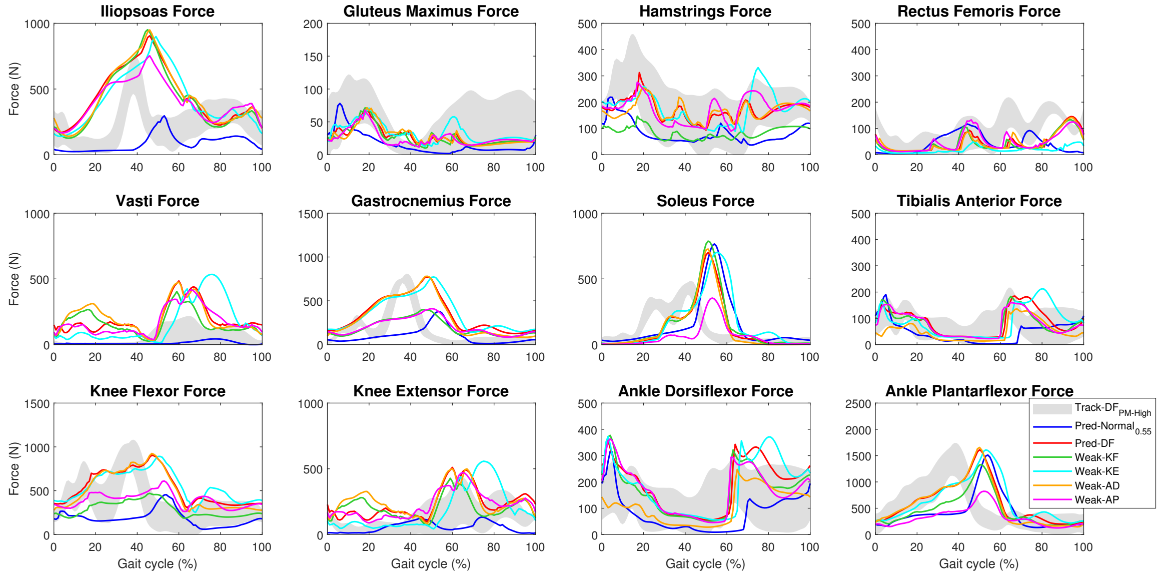

Figure S4. Influence of altered muscle–tendon parameters on Track-DF$_{\text{PM-High}}$ (mean ± standard deviation), Pred-Normal$_{0.55}$, Pred-DF, and Weak gait (ipsilateral major muscle forces). All simulations were performed at 0.55 m/s.

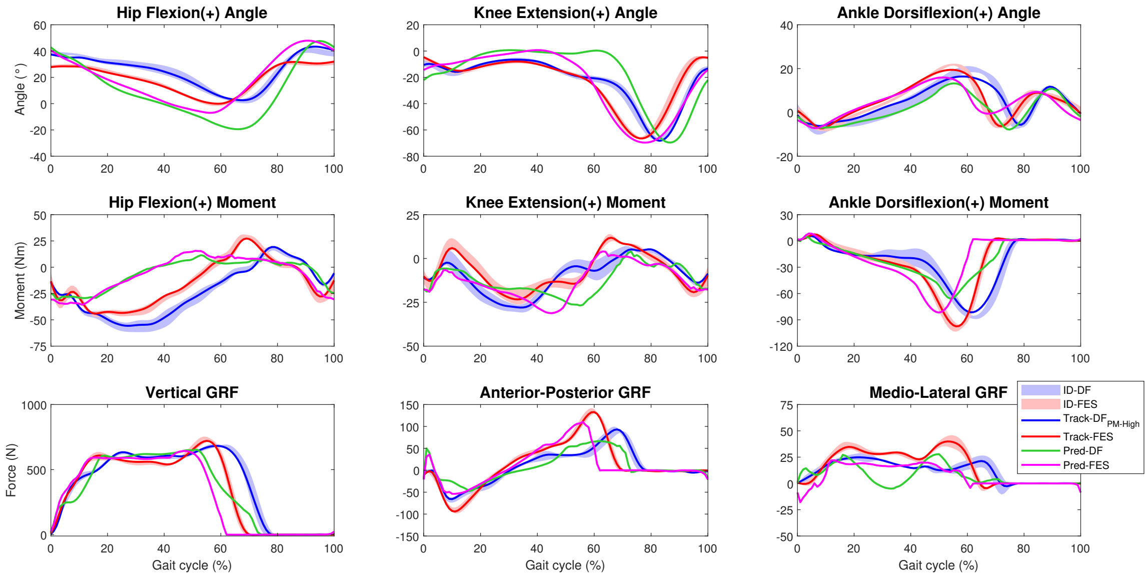

Figure S5. Effect of FES on ID (mean ± standard deviation), tracking, and prediction DF gait (contralateral hip, knee, and ankle angles, moments, and GRF). Ipsilateral results are presented in Fig. 4 (main document).

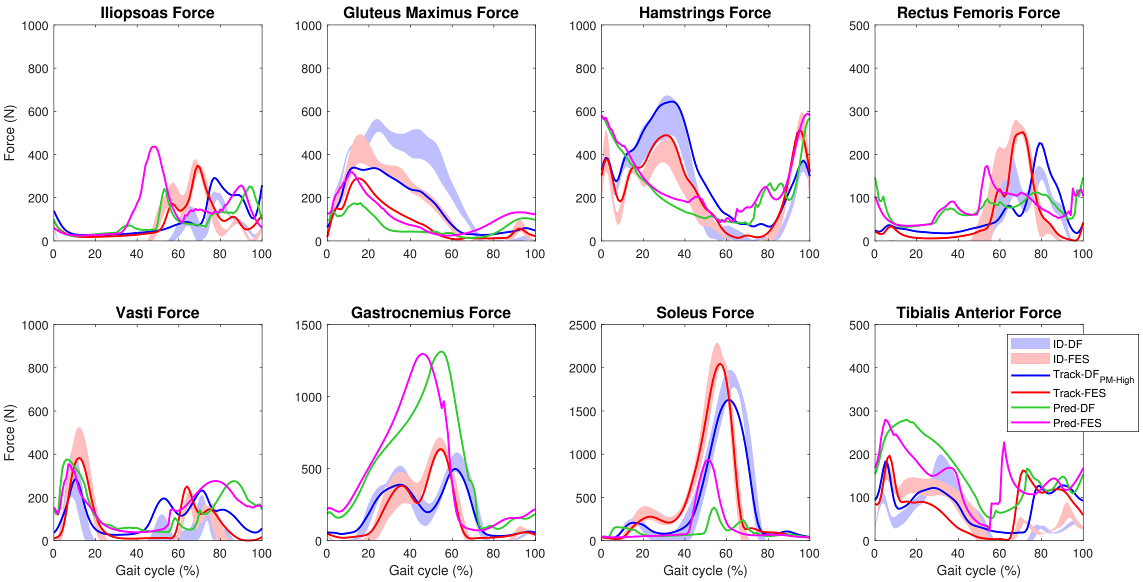

Figure S6. Effect of FES on ID (mean ± standard deviation), tracking, and prediction DF gait (contralateral major muscle forces). Ipsilateral results are presented in Fig. 5 (main document).

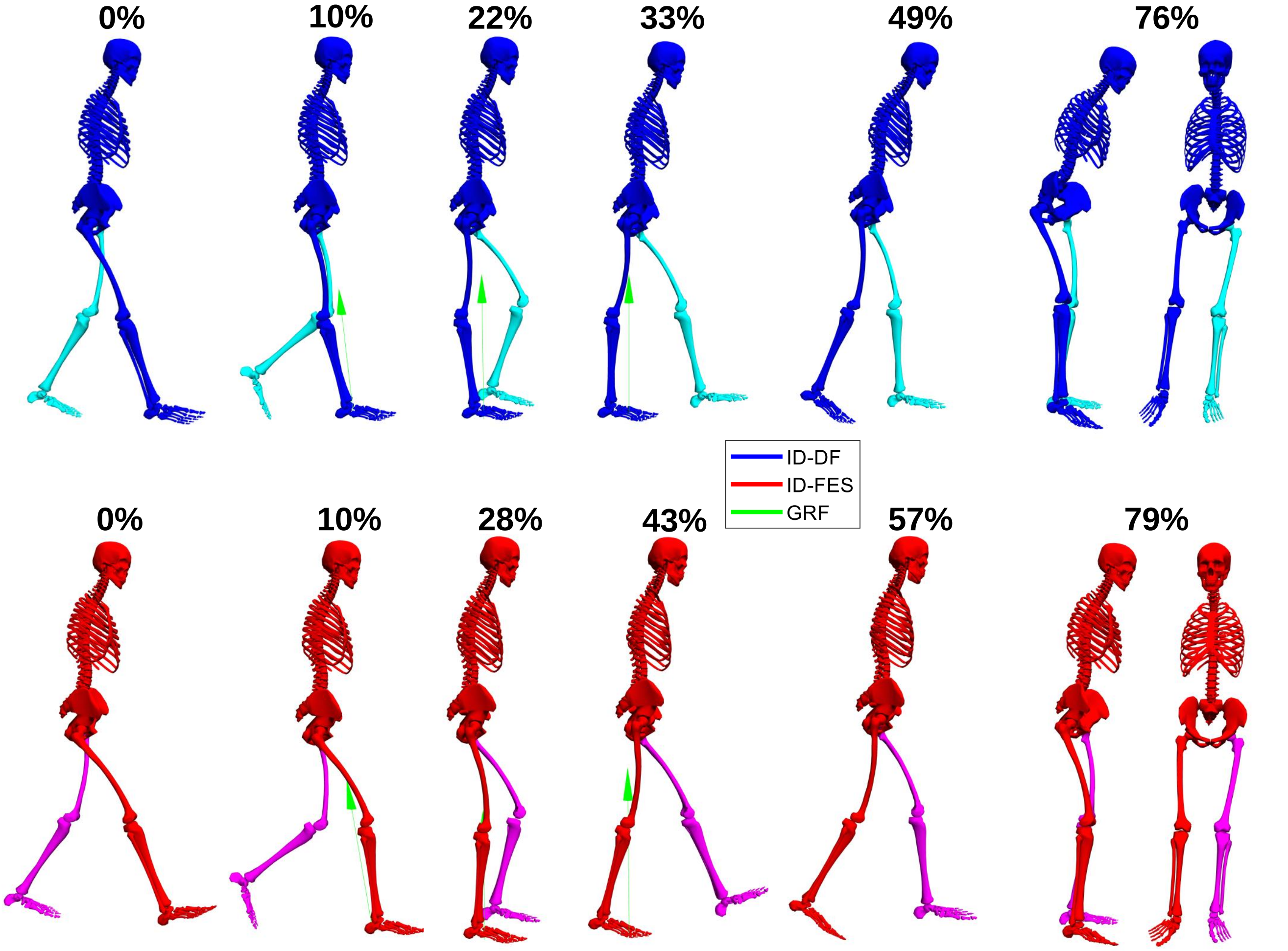

Figure S7. Effect of FES on ID gait pattern in a representative trial for DF and FES conditions. Views in the sagittal plane and frontal plane (last column) are shown. The green arrow depicts the experimental resultant GRF vector for the ipsilateral leg. Contralateral leg is shown in different color. See also Supplementary Video S1.

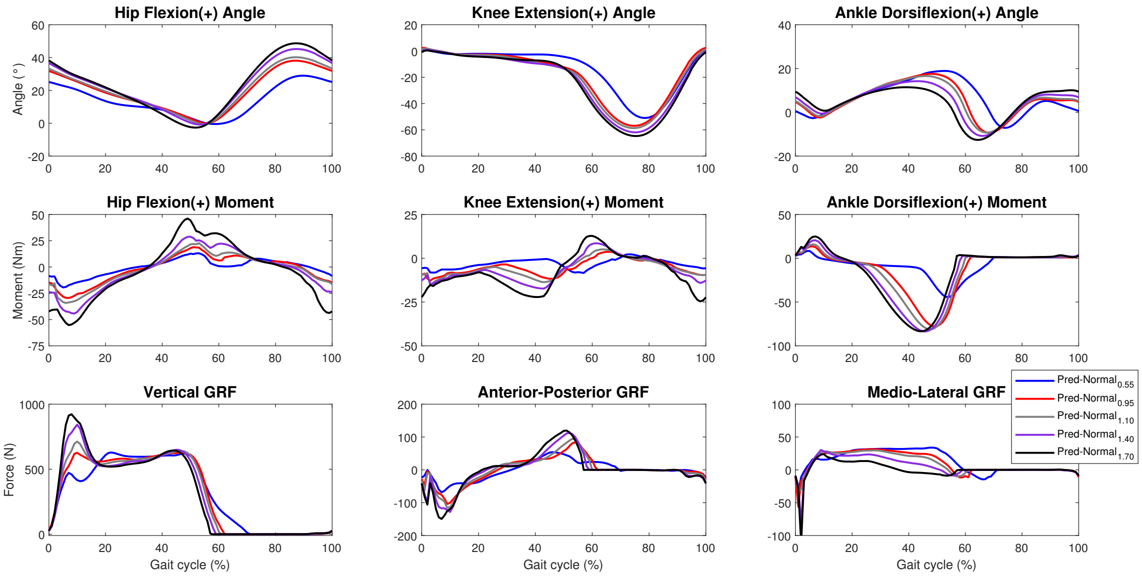

Figure S8. Influence of gait speed on Pred-Normal gait (ipsilateral hip, knee, and ankle angles, moments, and GRF).

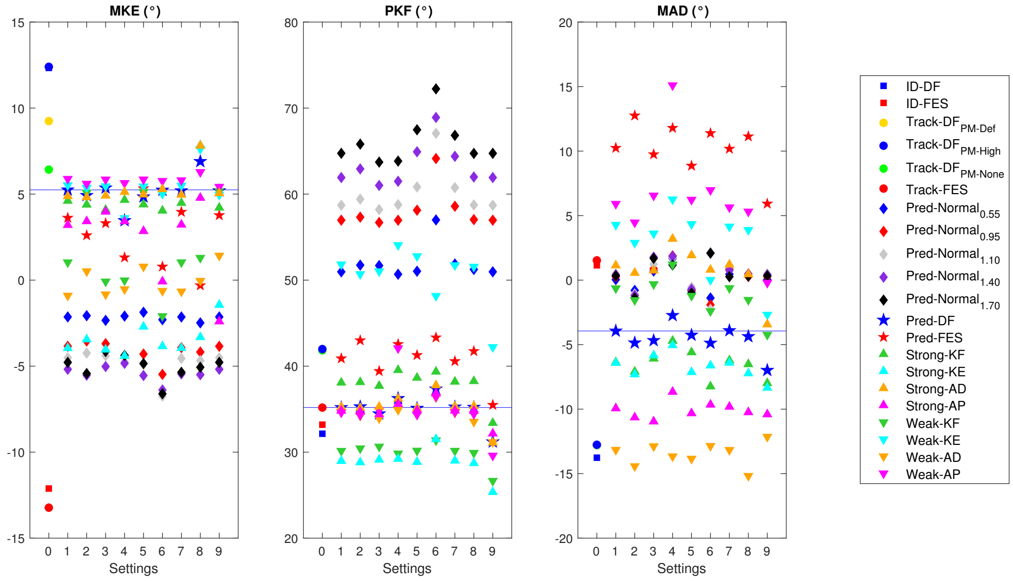

Figure S9. Influence of different settings on mean knee extension (MKE), peak of knee flexion (PKF), and mean ankle dorsiflexion (MAD). Only values for the ipsilateral leg are shown. The horizontal blue lines indicate the values for Pred-DF in Setting 1.

References

1. Falisse, A. *et al.* Physics-based simulations to predict the differential effects of motor control and musculoskeletal deficits on gait dysfunction in cerebral palsy: A retrospective case study. *Front. Hum. Neurosci.* **14**, 40 (2020).
2. De Groote, F., Kinney, A. L., Rao, A. V. & Fregly, B. J. Evaluation of direct collocation optimal control problem formulations for solving the muscle redundancy problem. *Ann. Biomed. Eng.* **44**, 2922–2936 (2016).
3. Falisse, A. *et al.* Rapid predictive simulations with complex musculoskeletal models suggest that diverse healthy and pathological human gaits can emerge from similar control strategies. *J. R. Soc. Interface* **16**, 20190402 (2019).
4. Bhargava, L. J., Pandy, M. G. & Anderson, F. C. A phenomenological model for estimating metabolic energy consumption in muscle contraction. *J. Biomech.* **37**, 81–88 (2004).
5. Uchida, T. K., Hicks, J. L., Dembia, C. L. & Delp, S. L. Stretching your energetic budget: How tendon compliance affects the metabolic cost of running. *PLOS ONE* **11**, e0150378 (2016).
